# Genetic Evidence for Function of the bHLH-PAS Protein Gce/Met As a Juvenile Hormone Receptor

**DOI:** 10.1371/journal.pgen.1005394

**Published:** 2015-07-10

**Authors:** Marek Jindra, Mirka Uhlirova, Jean-Philippe Charles, Vlastimil Smykal, Ronald J. Hill

**Affiliations:** 1 Biology Center, Czech Academy of Sciences, Ceske Budejovice, Czech Republic; 2 Commonwealth Scientific and Industrial Research Organization (CSIRO), Food and Nutrition Flagship, North Ryde, New South Wales, Australia; 3 Institute for Genetics and Cologne Excellence Cluster on Cellular Stress Responses in Aging-Associated Diseases (CECAD), University of Cologne, Cologne, Germany; 4 Centre des Sciences du Gout et de l’Alimentation (CSGA), CNRS 6265, INRA 1324, Université Bourgogne-Franche-Comté, Dijon, France; 5 Department of Molecular Biology, Faculty of Science, University of South Bohemia, Ceske Budejovice, Czech Republic; University of California, Riverside, UNITED STATES

## Abstract

Juvenile hormones (JHs) play a major role in controlling development and reproduction in insects and other arthropods. Synthetic JH-mimicking compounds such as methoprene are employed as potent insecticides against significant agricultural, household and disease vector pests. However, a receptor mediating effects of JH and its insecticidal mimics has long been the subject of controversy. The bHLH-PAS protein Methoprene-tolerant (Met), along with its *Drosophila melanogaster* paralog *germ cell-expressed* (Gce), has emerged as a prime JH receptor candidate, but critical evidence that this protein must bind JH to fulfill its role in normal insect development has been missing. Here, we show that Gce binds a native *D*. *melanogaster* JH, its precursor methyl farnesoate, and some synthetic JH mimics. Conditional on this ligand binding, Gce mediates JH-dependent gene expression and the hormone's vital role during development of the fly. Any one of three different single amino acid mutations in the ligand-binding pocket that prevent binding of JH to the protein block these functions. Only transgenic Gce capable of binding JH can restore sensitivity to JH mimics in *D*. *melanogaster Met*-null mutants and rescue viability in flies lacking both Gce and Met that would otherwise die at pupation. Similarly, the absence of Gce and Met can be compensated by expression of wild-type but not mutated transgenic *D*. *melanogaster* Met protein. This genetic evidence definitively establishes Gce/Met in a JH receptor role, thus resolving a long-standing question in arthropod biology.

## Author Summary

Juvenile hormones (JHs) play critical roles in the development of arthropods, comprising half the animal biomass of the oceans and over a million insect species, which have an enormous impact on ecosystems, agriculture (pollinators and pests) and health of mankind (disease vectors). Despite decades of research, a receptor for these unique sesquiterpenoid hormones has remained elusive. Here, we provide definitive genetic evidence establishing that the essential biological function of the Gce/Met protein during insect development is critically dependent on its ability to bind JH, in effect functionally defining a JH receptor. Unequivocal identification of a JH receptor has profound implications for our understanding of arthropod biology. It also defines a molecular target for development of environmentally friendly, safer insecticides.

## Introduction

Arthropods possess unique sesquiterpenoid hormones, represented by the juvenile hormones (JHs) of insects [1] and their non-epoxidized precursor, methyl farnesoate (MF) in crustaceans [2,3]. JHs regulate insect metamorphosis, polymorphism and social caste determination, and adult reproductive physiology [1,4–6]. Although the sesquiterpenoid structure of JH was determined nearly five decades ago [7], a receptor for these important hormones has been notoriously difficult to identify. Non-peptide lipophilic hormones usually exert genomic effects by activating nuclear receptor proteins [8–10]. One insect member of the nuclear receptor family, Ultraspiracle (USP), has been proposed as a mediator of sesquiterpenoid action, initially of JH itself [11] and currently of MF [6,12–14]. USP is an appealing JH receptor candidate given its homology to the vertebrate retinoid X receptor (RXR) and an apparent level of similarity between JH and the RXR ligand, 9-*cis*-retinoic acid [15]. Moreover, USP is a subunit of the insect ecdysone receptor complex [10,16,17], thus providing a potential point where the steroid and JH signaling pathways might converge. Whether or not the putative hormone-binding pocket of USP is capable of biologically significant ligand binding is still debated [13,14,18,19].

Discovery of the *Methoprene-tolerant* (*Met*) gene that confers resistance to the JH analog insecticide methoprene in the fruit fly, *Drosophila melanogaster*, has provided an alternative JH receptor candidate [20]. Nonetheless, absence of obvious effects of *Met* mutations on *D*. *melanogaster* development argued against the JH receptor function of Met until knockdown of *Met* in the flour beetle, *Tribolium castaneum*, produced precocious metamorphosis phenotypes consistent with disrupted JH signaling [21]. Later, it was shown in *D*. *melanogaster* that simultaneous mutation of *Met* and deletion of its paralog, the *germ cell-expressed* (*gce*) gene, resulted in non-conditional lethality during the larva-pupa transition [22], corresponding to the lethal phase associated with deficiency of JH [22,23]. The *Met* and *gce* paralogs in *D*. *melanogaster* arose via gene duplication during "higher fly" evolution, whereas mosquitoes or beetles possess only a single gene [24]. Based mainly on evidence related to the position of introns, *gce* is ancestral to *Met* and, in spite of the nomenclature, *D*. *melanogaster gce* is more similar to the single *Met* genes found in other insects [24].

Met and Gce belong to the basic helix-loop-helix (bHLH)/Per-Arnt-Sim (PAS) family of transcription factors [25] that are distinctly different from nuclear receptor proteins. Although no bHLH-PAS protein has previously been proven to be a receptor for an authentic hormone, the vertebrate aryl hydrocarbon receptor (AhR) is a transcription factor activated by xenobiotics (e.g., dioxin), or by endogenous ligands such as tryptophan metabolites, binding to its PAS-B domain [26,27].

Like JH, Gce/Met is unique to arthropods, and thus may have evolved to mediate JH signaling in insects, crustaceans, and other related taxa. *In vitro*, the Met proteins from *D*. *melanogaster* [28,29], *T*. *castaneum* [29], and the *Aedes aegypti* mosquito [30] bind native JH (JH III) with nanomolar affinities. Specific mutations within the PAS-B domain of *T*. *castaneum* or *A*. *aegypti* Met preclude this JH binding [29,30]. JH induces Met to bind to another bHLH-PAS protein Taiman (Tai), also known as FISC or SRC [29,31–33]. The resulting complex binds JH-response DNA motifs and activates target gene transcription [30–35]. Similarly to Met, Tai has also been shown to mediate effects of JH on metamorphosis [36] and reproduction [37,38] in some insects. The *D*. *melanogaster* Met and Gce proteins interact with a chaperone Hsp83, which facilitates nuclear import of Met and expression of JH-induced genes such as *Krüppel homolog 1* (*Kr-h1*) [34]. Most recently, Met and Gce were shown to mediate the effect of the JH precursor MF, which has been established as a circulating hormone in *D*. *melanogaster* [39].

Taken together, the above results favor Gce/Met as a JH receptor candidate. However, to establish conclusively that Gce/Met is a JH receptor, it must also be demonstrated that binding of the hormone is a necessary condition for functioning of the candidate receptor *in vivo*, during normal insect development. This study employs the power of *Drosophila* genetics to provide this critical missing evidence. It shows that transgenic Gce or Met proteins restore the natural sensitivity to JH mimics in the *Methoprene-tolerant* mutants and rescue the non-conditionally lethal *Met gce* double-mutant flies as long as their JH-binding pocket is intact.

## Results and Discussion

### Gce and Tai activate transcription in response to JH III, synthetic JH mimics, and MF

**Fig 1:**
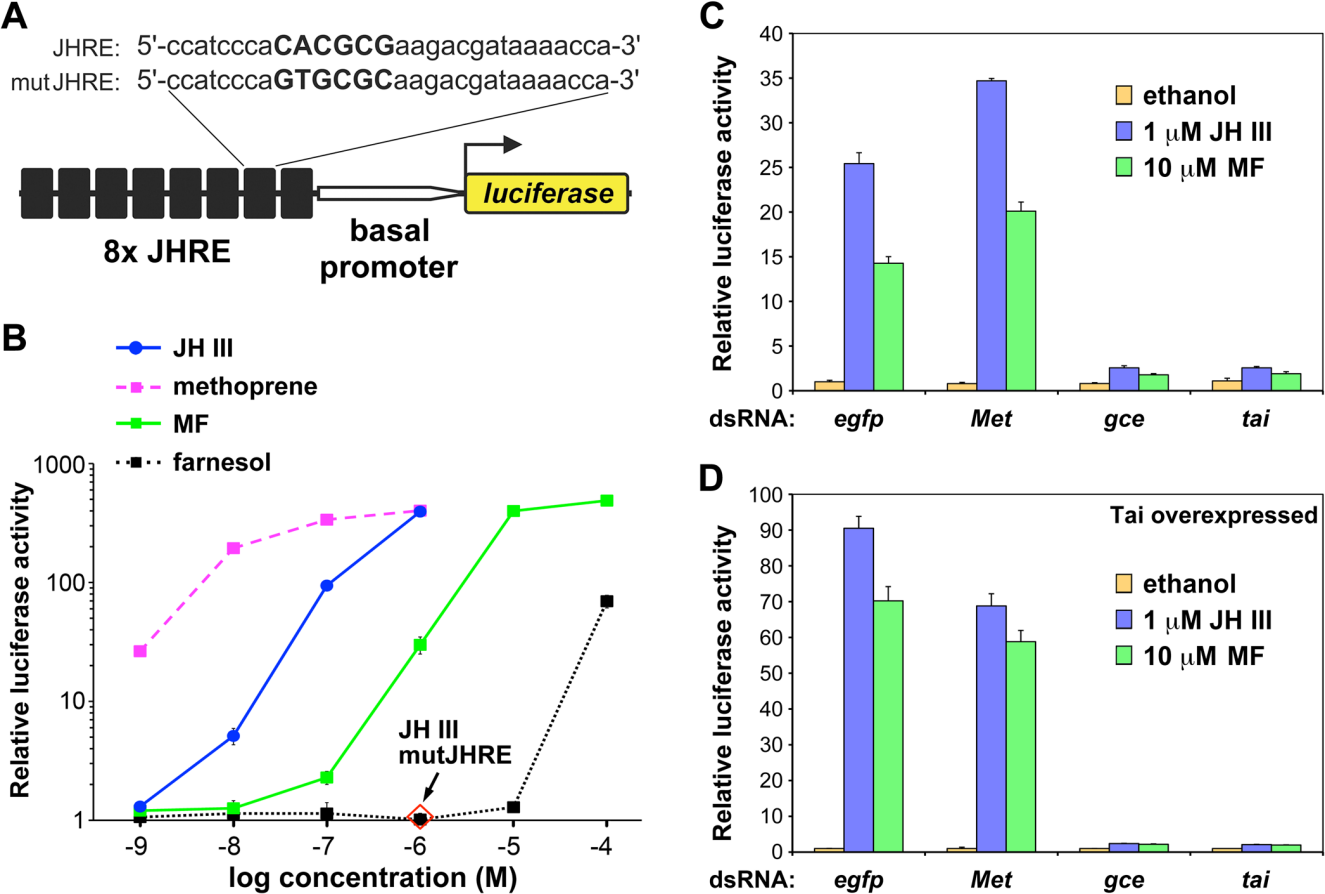
Transcriptional activation by JH III, methyl farnesoate (MF), and methoprene through Gce in *Drosophila* S2 cells. (A) A luciferase reporter construct carrying 8 copies of a JH response element (JHRE). The JHRE and the basal promoter derive from the *Aedes aegypti early trypsin* gene [30,31]. The core (capital letters) of the JHRE was mutated to produce mutJHRE. (B) S2 cells responded to the indicated compounds by activating the JHRE-luc reporter, but not mutJHRE-luc (1 μM JH III). Farnesol is a biologically inactive control. (C-D) Activation of JHRE-luc by 1 μM JH III or 10 μM MF relative to basal activity (ethanol) required both Gce and Tai as revealed by dsRNA-mediated knockdown of either protein; *egfp* dsRNA served as a control. Co-transfection with a Tai-expressing plasmid enhanced the Gce-dependent activation by JH III and MF (D). Data were normalized to *Renilla* luciferase activity and plotted as mean ± SD (n = 3).

The *D*. *melanogaster* S2 cell line expresses endogenous mRNAs encoding both Met and Gce paralogs and their single partner protein Tai (S1 Fig). We initially tested whether Met, Gce and Tai mediated ligand-dependent transcriptional activation in the S2 cells. A luciferase reporter JHRE-luc was constructed using eight tandem copies of a JH-response element (JHRE) from the *A*. *aegypti early trypsin* gene [30,31] (Fig 1A). JHRE-luc was activated by a native JH (JH III), the JH mimic methoprene, and by MF in a dose-dependent manner (Fig 1B). Mutation of the JHRE inhibited the response to JH III (Fig 1B). RNAi-mediated knockdown of either *tai* or *gce* but not of *Met* prevented JH III or MF from inducing JHRE-luc (Fig 1C). Expression of additional Tai enhanced this hormone-dependent activation, again in a manner dependent primarily on *gce* and *tai* (Fig 1D). Similar results were obtained utilizing pyriproxyfen, a potent JH mimic of distinct, pyridine-based chemical structure [40] (S2 Fig).

The observation that Gce and Tai were required for activation of JHRE-luc by MF (Fig 1C and 1D), is consistent with a previous finding that MF activated transcription through an ortholog of Gce/Met from the silkworm, *Bombyx mori* [29,31–33] and the recent finding that this natural JH precursor is a circulating hormone in *D*. *melanogaster* [39].

### Gce binds JH and its agonists including MF

**Fig 2:**
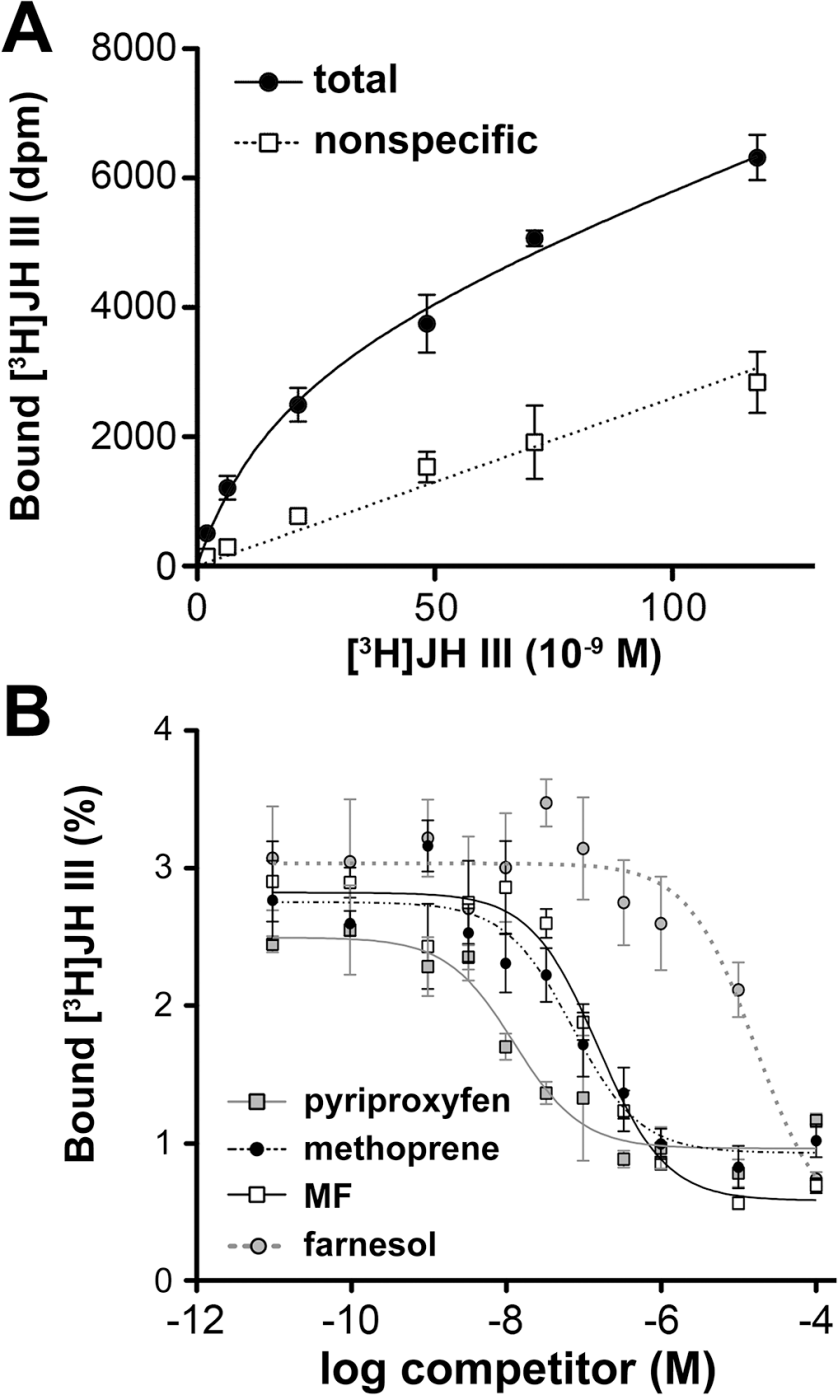
Binding of Gce by JH III, methyl farnesoate (MF), pyriproxyfen, and methoprene. (A) Gce translated *in vitro* was incubated with [^3^H]JH III in the absence (total binding) or presence (nonspecific binding) of a 100-fold molar excess of unlabeled JH III. Data are mean ± SD (n = 3); the calculated *K*_d_ is 19.3 ± 4.5 nM. (B) Competition binding assay: Gce was incubated with 2 pmol of [^3^H]JH III and increasing concentrations of the indicated compounds. The data (mean ± SEM from 3–5 experiments) indicated *K*_i_ values of 5.7 ± 2.8 nM for pyriproxyfen, 45.8 ± 28.9 nM for methoprene, 87.9 ± 22.2 nM for MF, and 8.1 ± 2.4 μM for farnesol (a biologically inactive control).

As the effect of JH in the S2 cell-based assay was essentially mediated by Tai and Gce, we examined the ability of the Gce protein *in vitro* to bind the activating ligands. [^3^H]JH III bound to Gce with a *K*_d_ of 19.3 ± 4.5 nM (Fig 2A), an affinity within the physiological hormone range [13]. Following on from the reporter gene activation data (Fig 1B), methoprene, pyriproxyfen, and MF all effectively competed with [^3^H]JH III for binding to Gce (Fig 2B), consistent with both JH mimics and MF acting as JH receptor agonists. Similarly to binding affinities previously determined for the PAS-B domain of *T*. *castaneum* Met [29], pyriproxyfen was the strongest competitor for binding to Gce, followed by methoprene and MF (Fig 2B). The higher potency of methoprene to activate JHRE-luc, relative to JH III (Fig 1B) may be explained by the fact that the synthetic insecticide is chemically and biologically more stable than JH III. Due to marginal levels of total [^3^H]JH III bound to the *in-vitro* translated *D*. *melanogaster* Met protein [29], we were unable to determine the ligand-binding affinities for Met.

The activation and binding of Gce by MF is significant, as this circulating JH precursor prevails over JH III in *D*. *melanogaster* larvae [13,39] and exerts its own hormonal function [39]. Interestingly, MF has been reported to bind *D*. *melanogaster* USP with a high affinity (*K*_d_ = 40 nM) [12], comparable to the *K*_i_ of 87.9 nM we observed for MF binding to Gce (Fig 2B). USP has therefore been proposed as an intracellular MF receptor [6,13,14]. However, in agreement with genetic evidence [39], our RNAi data (Fig 1C and 1D) clearly show that Gce and Tai are essential for MF to induce expression of the JHRE-dependent reporter and thus act as a MF receptor.

**Fig 3:**
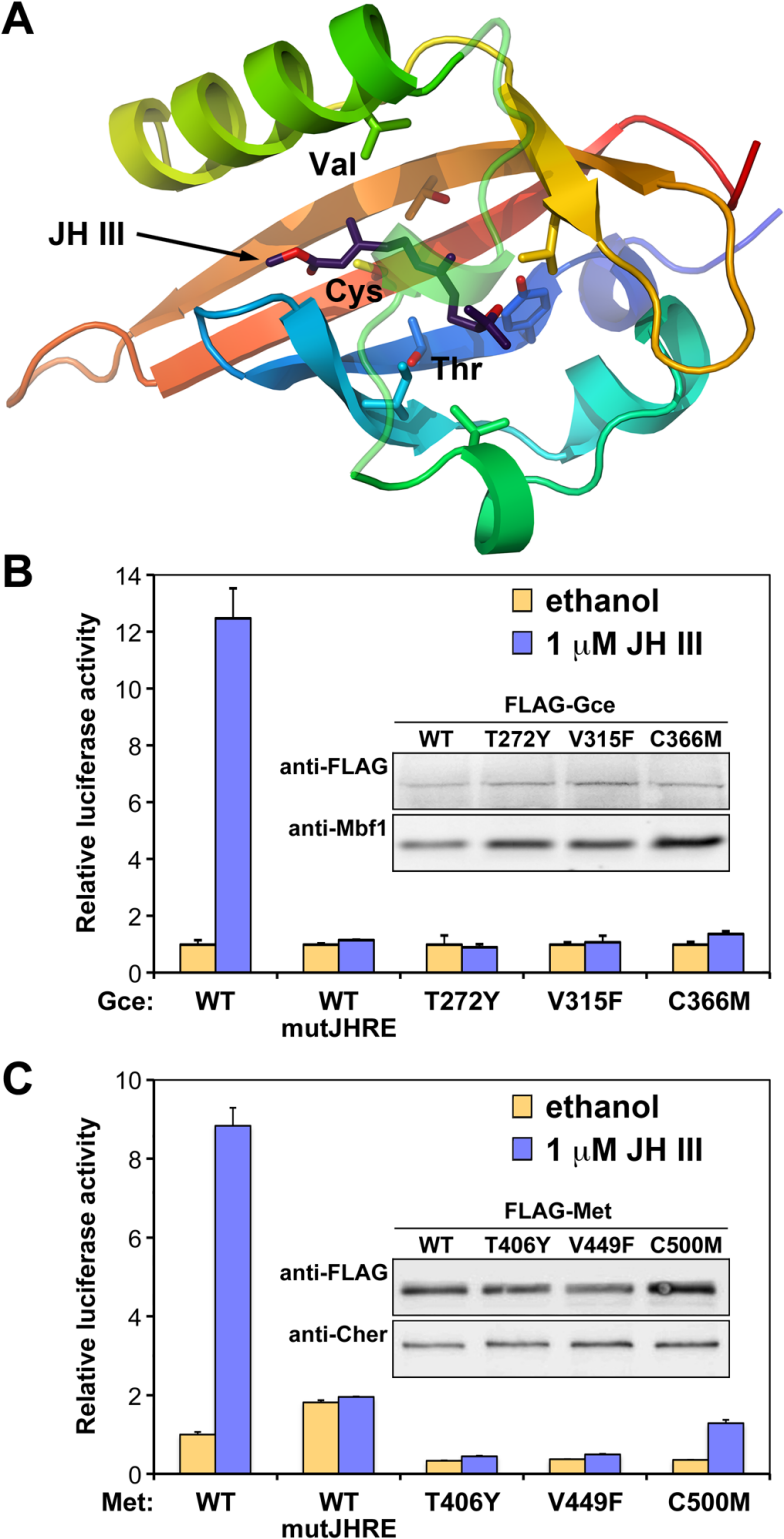
Gce and Met mutated in their JH-binding domains are incapable of activating transcription. (A) Positions of three conserved amino acids important for binding of JH III based on our model of the *T*. *castaneum* Met PAS-B domain [29]. (B) Only wild-type Gce (WT) capable of binding JH III (S3 Fig) activated the JHRE-luc reporter in S2 cells. (C) Similar results were obtained for Met, which also lost its ability to activate JHRE-luc in response to JH III when its PAS-B domain was mutated at the corresponding conserved residues. In both experiments (B and C), the endogenous *gce* and *Met* were suppressed by RNAi. Data were normalized to *Renilla* luciferase activity and plotted as mean ± SD (n = 3). The WT and mutated Gce and Met variants were all stable as detected on immunoblots (insets) using their FLAG tags; antibody against the Mbf1 or Cheerio proteins served as controls.

MF is a "juvenile hormone" of crustaceans, where it promotes reproductive maturation and specific developmental events [41,42]. Interestingly, similar to JH in insects [29,31–33], MF has been shown to stimulate interaction between Met and Tai/SRC orthologs from the cladoceran crustaceans, *Daphnia pulex* and *D*. *magna* [43]. Moreover, when a threonine residue in the PAS-B domain of *Daphnia* Met was replaced with valine that occurs in the corresponding position critical for JH III binding in insects, namely V315 in *D*. *melanogaster* Gce (Fig 3A and 3B) or V297 in *T*. *castaneum* Met [29], the *Daphnia* Met protein became more responsive to JH III, without losing its responsiveness to MF [43]. Together with our current findings, this recent evidence suggests that Gce/Met has evolved as a receptor for sesquiterpenoid hormones in a common ancestor of crustaceans and insects.

### Gce/Met requires ligand binding to induce transcription in response to JH

To determine whether Gce required direct binding of JH for its function, we individually mutated three amino acids (T272Y, V315F, and C366M) in the ligand-binding pocket of Gce PAS-B (Fig 3A). The same substitutions of the corresponding residues have been shown to abolish binding to JH III in the Met proteins from *T*. *castaneum* [29] and *A*. *aegypti* [30]. As expected, all three mutated Gce proteins lost the ability to bind [^3^H]JH III *in vitro* (S3 Fig), indicating that these conserved T, V and C residues are critical for hormone binding by *D*. *melanogaster* Gce.

To test whether binding of JH was necessary for Gce to activate transcription, FLAG epitope-tagged wild-type (FLAG-Gce^WT^) or mutated (FLAG-Gce^T272Y^,-Gce^V315F^, and-Gce^C366M^) proteins were expressed in S2 cells, in which the endogenous Gce and Met were suppressed by RNAi. Clearly, only FLAG-Gce^WT^ responded to JH III to activate the JHRE-luc reporter, whereas the three Gce variants incapacitated for hormone binding did not (Fig 3B). The wild-type and mutated Gce proteins all appeared to be stable in the S2 cells (Fig 3B, inset).

Although the endogenous Met protein did not appear to play a major role in the JH-dependent activation of JHRE-luc in the S2 cell line (Fig 1C and 1D), *D*. *melanogaster* Met could in fact substitute for Gce in this reporter assay when transfected to the cells (Fig 3C). The S2 cells were again subjected to RNAi-mediated depletion of the endogenous Met and Gce proteins but not of the added Met protein that was expressed from a synthetic DNA construct. Like Gce, Met but not its mutated versions, mediated induction of JHRE-luc by JH III (Fig 3C). Importantly, the functional JHRE-luc reporter was not activated by Met that had been mutated in its PAS-B domain with individual substitutions T406Y, V449F, and C500M that correspond to the T272Y, V315F, and C366M mutations in Gce (Fig 3C). These mutations did not lead to degradation of Met (Fig 3C, inset). Although we have been unable to directly confirm the effect of these mutations on the ligand-binding activity of *D*. *melanogaster* Met, it is most likely that they prevent JH III binding just as equivalent substitutions of these highly conserved residues do in Gce (S3 Fig) and in the Met proteins from *T*. *castaneum* [29] and *A*. *aegypti* [30]. These results strongly suggest that the JH-binding capacity is required for the normal function of *D*. *melanogaster* Met.

### Expression of an endogenous JH-response gene relies on the JH-binding activity of Gce

In *D*. *melanogaster* the *Met* and *gce* genes reside on the X chromosome and their simultaneous loss in females that are homozygous or males that are hemizygous for the *Met* ^*27*^ and *gce* ^*2*.*5k*^ null alleles is lethal at the onset of pupation [22]. The *Met* ^*27*^ *gce* ^*2*.*5k*^ double mutants are known to express reduced mRNA levels of the *Kr-h1* gene, which is a direct target of Gce/Met [22,34,39]. In order to demonstrate that the JH-binding capacity of Gce is important *in vivo* for transcription of this relevant JH-response gene, we expressed the wild-type and mutated forms of Gce using the ubiquitous *armadillo-Gal4* driver (*arm-Gal4*) in the *Met* ^*27*^ *gce* ^*2*.*5k*^ background. To avoid the lethal phase in this strain, we examined *Kr-h1* expression in *Met* ^*27*^ *gce* ^*2*.*5k*^ */Y* male larvae that were selected and genotyped during mid-third instar. The equal performance of the Gce variants was ensured by inserting all transgenic *UAS-gce* constructs into the same genetic locus [44].

**Fig 4:**
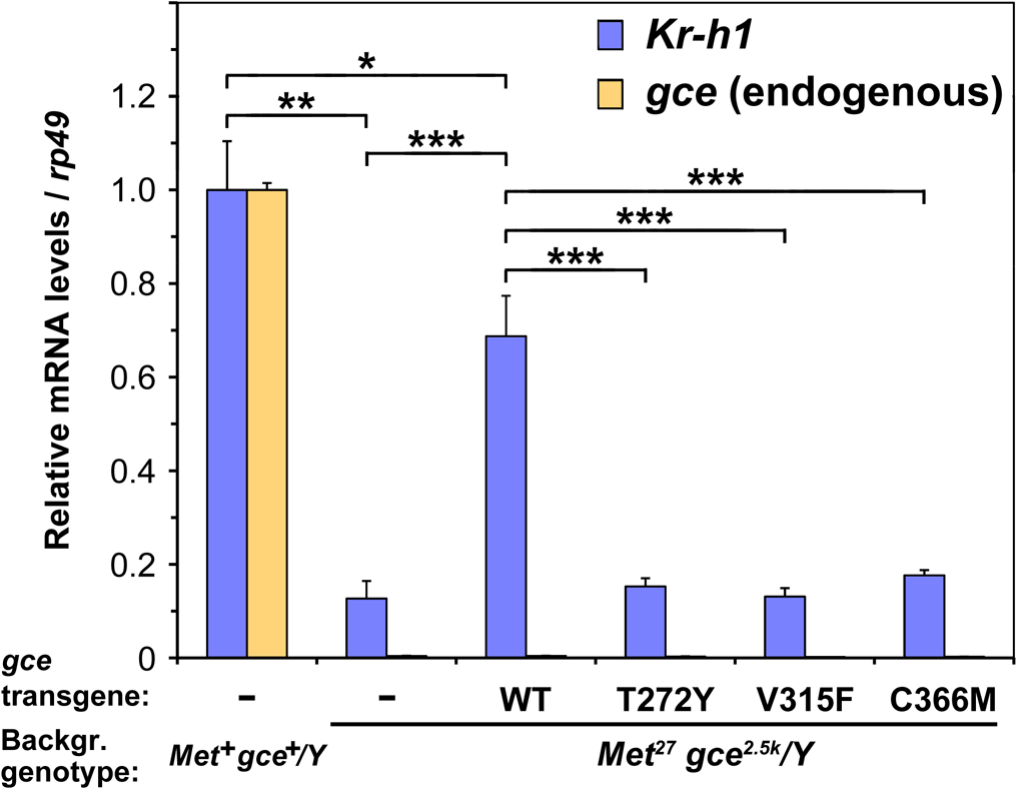
The ligand-binding capacity of Gce is required for normal expression of the direct JH-response gene *Kr-h1 in vivo*. Reduction in *Kr-h1* mRNA levels in *Met* ^*27*^ *gce* ^*2*.*5k*^ mutant larvae can be compensated by transgenic expression of the functional Gce protein but not by any of its mutated forms, incapable of binding JH III (S3 Fig). Balanced *Met* ^*27*^ *gce* ^*2*.*5k*^ */FM7c; arm-Gal4* females were crossed with males bearing the *UAS-gce* transgenes. The male progeny were selected as mid-third instar larvae and genotyped by PCR detecting the *gce* ^*2*.*5k*^ deletion [22] to distinguish *Met* ^*27*^ *gce* ^*2*.*5k*^ */Y* from *Met* ^*+*^ *gce* ^*+*^ */Y* siblings carrying the *FM7c* balancer chromosome. Groups of four larvae were subjected to qRT-PCR with primers detecting the endogenous *Kr-h1α* and *gce* transcripts (S2 Table). Values relative to mRNA levels in *Met* ^*+*^ *gce* ^*+*^ */Y* controls (set to 1) are mean ± SD from three biological replicates for each genotype, five replicates for Gce^WT^. The significance levels of differences determined by *t*-test were *P* < 0.02 (*), *P* = 0.0002 (**), and *P* < 0.0002 (***).

Consistent with previous reports [22,34,39], we observed reduced *Kr-h1* levels in *Met* ^*27*^ *gce* ^*2*.*5k*^ mutants (Fig 4) albeit the difference was less dramatic, likely due to the earlier stage of our animals at which *Kr-h1* expression is lower and less dependent on JH [45]. Addition of the transgenic Gce^WT^ protein to the *Met* ^*27*^ *gce* ^*2*.*5k*^ background significantly augmented *Kr-h1* expression near levels occurring in *Met* ^*+*^ *gce* ^*+*^ sibling male larvae (Fig 4). In contrast, the *Kr-h1* transcript remained low when any of the three mutated forms of Gce were expressed (Fig 4). Therefore, only when capable of binding its hormonal ligand (S3 Fig), Gce could compensate for the missing endogenous receptor proteins in restoring the normal expression of their target gene. As the function of *Kr-h1* is essential for *D*. *melanogaster* to complete the prepupal stage [45], compromised *Kr-h1* expression may be contributing to the lethality resulting from the absence of Gce and Met.

### The ligand-binding capacity of Gce/Met is necessary for the normal response of *Drosophila* to JH mimics

To further investigate the receptor function of Gce *in vivo*, we tested the relationship of JH binding to the phenomenon of "methoprene tolerance"–the insecticide resistance phenotype for which the *D*. *melanogaster Met* mutants were originally isolated and named [20]. Strains singly mutant either for *Met* or, to a lesser extent *gce*, resist doses of JH mimics that kill flies possessing both wild-type genes [20,22,46,47]. It has been shown that ubiquitous expression of a *gce* ^*+*^ transgene using the Gal4/UAS system is sufficient to reinstate sensitivity to methoprene in the *Met* ^*27*^ null mutants [46].

**Fig 5:**
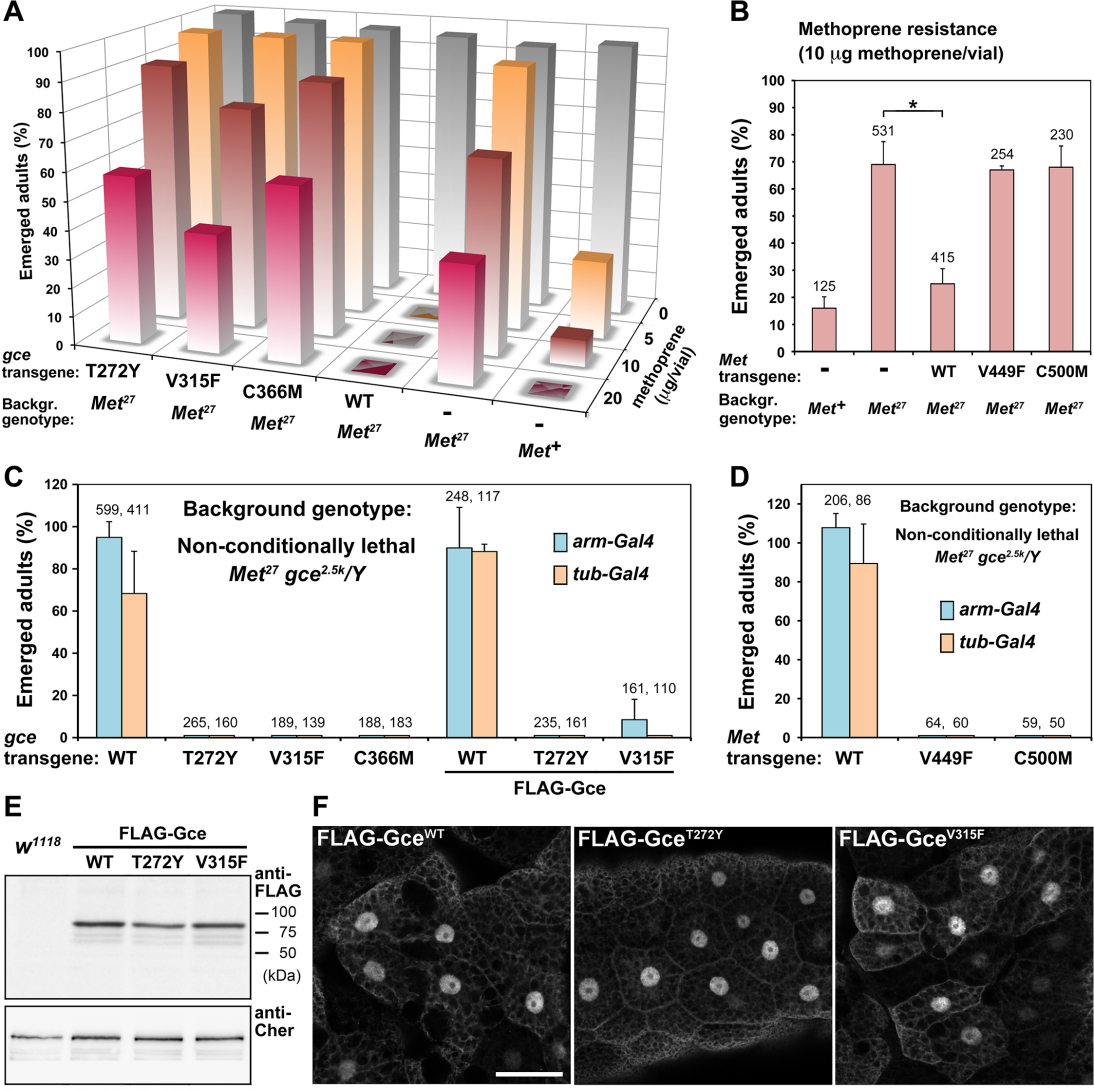
The capacity to bind JH is essential for Gce/Met function *in vivo*. (A-B) *Met* ^*27*^ mutants tolerate methoprene better than *Met* ^*+*^ control flies [47] or *Met* ^*27*^ flies expressing transgenic Gce^WT^ or Met^WT^ proteins. *Met* ^*27*^ */Y* males carrying the indicated *UAS-gce* or *UAS-Met* transgenes were mated to *Met* ^*27*^ *; arm-Gal4* females, and the F1 progeny was fed methoprene. In the presence of methoprene, Gce^WT^ totally blocked adult development and Met^WT^ significantly (*; *P* < 0.0003) reduced survival relative to the *Met* ^*27*^ strain, whereas mutated Gce or Met did not have this effect. Values are per cent average numbers of emerged adults relative to total numbers of pupated animals. Each column represents 200–430 animals (or the indicated numbers in B) counted in 2–3 independent trials. (C-D) Balanced *Met* ^*27*^ *gce* ^*2*.*5k*^ */FM7c; arm-Gal4* (or *tub-Gal4*) females were crossed with males bearing the *UAS-gce* or *UAS-Met* transgenes, and emerged *Met* ^*27*^ *gce* ^*2*.*5k*^ */Y* adult males were scored relative to their *FM7c/Y* (*Met* ^*+*^ *gce* ^*+*^) siblings (1:1 ratio was considered 100% rescue). Non-conditionally lethal *Met* ^*27*^ *gce* ^*2*.*5k*^ */Y* males were rescued to adulthood by transgenic Gce^WT^, FLAG-Gce^WT^, and Met^WT^ proteins but not by their mutated versions except for a few flies rescued by one randomly inserted FLAG-Gce^V315F^ construct expressed under *arm-Gal4* (C). Data are mean ± SD; numbers of all scored F1 males are given above columns. (E) FLAG-Gce WT and mutated proteins were stable when expressed *in vivo* as shown on an immunoblot (anti-FLAG antibody) from adult flies. The Cheerio (Cher) protein served as a control. (F) FLAG-Gce WT and mutated proteins were detected with the anti-FLAG antibody in the nuclei of larval fat body cells. Bars, 50 μm.

We took this approach with our Gce^WT^, Gce^T272Y^, Gce^V315F^, and Gce^C366M^ transgenic constructs. When expressed under the *arm-Gal4* driver, only Gce^WT^ restored sensitivity to dietary methoprene in *Met* ^*27*^ homozygotes (Fig 5A). In fact, these *Met* ^*27*^ animals expressing Gce^WT^ became more sensitive to methoprene than *Met* ^*+*^ controls, reflecting a dominant effect of the additional Gce^WT^ protein. In contrast, *Met* ^*27*^ males and females expressing any of the three mutated Gce variants remained resistant and emerged as adults after feeding on methoprene (Fig 5A). Similar results were obtained with pyriproxyfen (S4 Fig). Thus, the lethal action of the insecticidal JH mimics relies on the ligand-binding capacity of the transgenic Gce protein.

To obtain similar information for Met, we repeated this experiment with fly strains expressing the *D*. *melanogaster* wild-type Met protein or its mutated versions Met^V449F^ and Met^C500M^ (our initial attempt to transform flies with *UAS-Met* ^*T406Y*^ failed). Although the flies expressing Met^WT^ under the *arm-Gal4* driver did not become more sensitive than control *Met* ^*+*^ flies, their response to dietary methoprene significantly increased relative to the original *Met* ^*27*^ mutants or the same mutants carrying the Met^V449F^ and Met^C500M^ transgenes (Fig 5B). Our data thus demonstrate that Gce and Met are mutual substitutes in rendering flies sensitive to exogenous JH mimics as long their ligand-binding pockets are unaffected by specific mutations.

### The capacity of Gce/Met to prevent lethality in *Met gce* double-mutant flies depends on hormone binding

The non-conditional lethality of the *Met* ^*27*^ *gce* ^*2*.*5k*^ double-mutants can be rescued with transgenic constructs providing either *Met* ^*+*^ or *gce* ^*+*^ function, thus reflecting partial redundancy between Met and Gce [22]. This genetic rescue offers an ideal system to answer the ultimate question as to whether Gce/Met requires its JH-binding capacity to sustain normal development of the animal. Using two ubiquitous drivers, *arm-Gal4* and *α-tubulin* (*tub-Gal4*), and transgenic *UAS-gce* and *UAS-Met* constructs uniformly inserted to the *attP2* chromosomal site [44], we expressed the functional or mutated proteins in the *Met* ^*27*^ *gce* ^*2*.*5k*^ background. Indeed, expression of Gce^WT^ or Met^WT^ under both drivers rescued a major proportion of *Met* ^*27*^ *gce* ^*2*.*5k*^ */Y* hemizygous males to adulthood (Fig 5C and 5D). In striking contrast, the mutated Gce^T272Y^, Gce^V315F^, Gce^C366M^, Met^V449F^ or Met^C500M^ proteins did not allow any *Met* ^*27*^ *gce* ^*2*.*5k*^ */Y* adults to emerge (Fig 5C and 5D). Therefore, only Gce/Met with intact JH-binding function can substitute for the absence of both genes during normal development.

To examine whether Gce incapacitated for JH binding was stable *in vivo*, we expressed FLAG-tagged versions of Gce^WT^, Gce^T272Y^, and Gce^V315F^ in transgenic *D*. *melanogaster*. Again, only the functional but not the JH binding-deficient tagged protein provided a clear rescue of the *Met* ^*27*^ *gce* ^*2*.*5k*^ mutants (Fig 5C). Interestingly, marginal rescue of 7.5% of emerging adults was observed, albeit only with the *arm-Gal4* driver, with FLAG-Gce^V315F^ (Fig 5C), suggesting that this mutated protein might retain some residual functionality. The discrepancy between this weak effect and the total absence of rescue by untagged Gce^V315F^ (Fig 5C) might result from variable expression level of the FLAG-tagged construct that, unlike the untagged constructs, had been integrated to random loci rather than to the specific *attP2* site. Indeed, from three independent FLAG-Gce^V315F^ transgenic lines, only one showed the partial genetic rescue.

Importantly, all three FLAG-tagged Gce variants were detected on immunoblots from whole transgenic flies (Fig 5E), and all were observed primarily in the nuclei of larval fat body cells (Fig 5F), regardless of whether or not Gce was mutated to prevent binding of JH. Thus, the failure of mutated Gce to compensate for the loss of the endogenous Met and Gce proteins was more likely caused by inability to bind JH rather than by degradation or mislocalization of the mutated protein.

In conclusion, our study shows that the capacity of Gce, and most likely also of Met, to promote gene expression and sustain normal development requires direct hormone binding to the protein *in vivo*. The case that Gce/Met acts as a JH receptor in insects is now unequivocal. Establishment of the nature of this receptor resolves a central problem in arthropod endocrinology. The ability of Gce to respond to methyl farnesoate, the crustacean JH, suggests that the role of Gce/Met in sesquiterpenoid signaling predates the evolutionary separation of the hexapod and crustacean lineages. Furthermore, it is of interest that Gce/Met provides the first clear example of a bHLH-PAS protein acting as a receptor for a genuine animal hormone.

## Materials and Methods

### Vectors for Gce and Met protein expression

DNA sequences corresponding to the *D*. *melanogaster* Gce (amino acids 1–689; NCBI Reference Sequence NP_511160.1) and Met (amino acids 1–716; NCBI Reference Sequence NP_511126.2) were synthesized for optimal *D*. *melanogaster* codon usage to encode the Gce and Met wild-type (WT) and mutated (T272Y, V315F, C366M, T406Y, V449F, C500M) variants. For transcription/translation *in vitro*, these DNA fragments were cloned using the *Eco* RI and *Kpn* I restriction sites behind the T7 promoter in the *pK-Myc-C2* plasmid [48]. The same *gce* and *Met* DNA sequences were inserted under the *UAST* promoter in two different vectors for *D*. *melanogaster* transformation: *pTFW* (*Drosophila* Genomics Resource Center), in which Gce was N-terminally tagged with a FLAG epitope, and *pUASTattB* [49] that permitted integration of the *gce/Met* transgenes into the specific *attP2* chromosomal landing site [44].

### Ligand-binding assays

Racemic (RS) tritiated JH III (10–20 Ci mMol^-1^) was purchased from Perkin Elmer. Racemic JH III, pyriproxyfen, *trans*,*trans*-farnesol and methoprene were from Sigma-Aldrich, and (E,E)-methyl farnesoate (MF) from Echelon Biosciences. The WT, T272Y, V315F, and C366M variants of Gce were produced with the rabbit reticulocyte lysate TnT Quick Coupled transcription/translation system (Promega) using 400 ng of template plasmid per 50-μl reaction. Each reaction was divided into 15-μl aliquots that were assessed for binding of [^3^H]JH III using the dextran-coated charcoal (DCC) method as described previously [29]. The dissociation constant (*K*_d_) was determined for Gce^WT^ binding to [^3^H]JH III, and the *K*_i_ values for methoprene, pyriproxyfen, MF and farnesol were calculated from competition assays with the unlabeled compounds using GraphPad Prism 5.00 (GraphPad Software) as described [29].

### Luciferase reporter assays

A JH-responsive luciferase reporter (JHRE-luc) was generated using a JH response element (JHRE) (5'- CCATCCCACACGCGAAGACGATAAAACCA- 3') identified upstream of the *Aedes aegypti early trypsin* (*AaET*) gene [31]. A mutated version of this element (5'- CCATCCCAGTGCGCAAGACGATAAAACCA -3') was used to generate a negative-control mutJHRE-luc. DNA sequences were synthesized to include eight copies of either JHRE or mutJHRE, followed by a 140-bp minimal promoter of the *AaET* gene (nucleotides -77 to +63). These sequences were cloned to the *pGL4*.*17* vector containing the firefly (*Photinus pyralis*) *luc2* gene (Promega). *D*. *melanogaster* Schneider 2 (S2) cells were cultured in Shields and Sang M3 Insect Medium (Sigma-Aldrich) containing 8% of heat-inactivated fetal bovine serum (Life Technologies) at 25°C. For luciferase reporter assays, S2 cells were seeded in a 12-well plate containing 900 μl of medium per well, and cultured for 24 h. The JHRE-luc (or mutJHRE-luc) reporter plasmid (0.25 μg per well) was co-transfected with a *pCopia* plasmid (0.1 μg per well) encoding *Renilla* luciferase using the X-tremeGENE HP DNA Transfection Reagent (Roche). Where appropriate, the *D*. *melanogaster* Tai protein was expressed from a *pCMA* plasmid (0.25 μg per well) containing *tai* cDNA [31,50]. Expression of either the wild-type or mutated FLAG-tagged Gce and Met variants was achieved by co-transfecting 0.25 μg of a *pTFW* vector carrying the respective *gce* or *Met* DNA sequence under the *UAST* promoter with 0.1 μg of a plasmid expressing the Gal4 transcription factor under a *D*. *melanogaster actin* promoter. The total DNA load per well was kept constant at 1 μg by inclusion of non-specific plasmid DNA. Following transfection, cells were incubated for 48 h and treated for another 12 h with JH III, methoprene, pyriproxyfen, MF or farnesol (all dissolved in ethanol). The cells were then processed with the Dual-Luciferase reporter assay system (Promega). Relative luciferase activity was measured using the Orion II microplate luminometer (Berthold Detection Systems) and data were normalized against *Renilla* luciferase activity.

### RNAi in S2 cells

*Met*, *gce* and *tai* cDNAs were obtained by reverse transcription of total *D*. *melanogaster* embryonic RNA, followed by PCR amplification with specific primer sets (S1 Table). The cDNA fragments were flanked with T7 promoter sequences to enable synthesis of double-stranded RNA (dsRNA) using T7 RNA polymerase (MEGAscript, Ambion). A 720-bp dsRNA derived from the *egfp* gene served as a control. To knock down *Met*, *gce*, and *tai* genes in S2 cells, 3 μg of dsRNA per well of a 12-well plate were added together with plasmid DNA in the transfection mixture. The dsRNA sequences targeting endogenous *gce* and *Met* did not interfere with expression of the Gce and Met (WT or mutated) proteins transfected with the *pTFW* vector, as those were encoded by synthetic DNA divergent from the endogenous DNA sequences. Moreover, *gce* dsRNA targeted an upstream region of the native *gce* transcript that did not overlap with the synthetic sequence included in the *pTFW-gce* constructs.

### mRNA quantification

Total RNA isolated from whole mid-third instar *D*. *melanogaster* larvae or S2 cells using the Trizol reagent (Life Technologies) was treated with TURBO DNase (Ambion), and 1.5 μg of RNA was reverse transcribed to cDNA (Superscript II, Life Technologies). Relative transcript levels were measured in a C1000 Thermal Cycler (Bio-Rad) using the iQ SYBR Green Supermix kit (Bio-Rad) using specific primer sets (S2 Table) and normalized against levels of the *ribosomal protein 49* (*rp49*) mRNA.

### *Drosophila* transgenesis

Targeted insertion of *gce* transgenes into the *attP2* landing site on the third chromosome (cytological position 68A4) was achieved using the bacteriophage ϕC31 integrase method [44]. The *pUASTattB* constructs containing the WT and mutated *gce* or *Met* sequences were injected into embryos of the *y w P{nos-ϕC31\int*.*NLS}X; P{CaryP}attP2* host strain (Genetic Services, Inc. or BestGene, Inc.). Several independent transgenic lines for expression of the FLAG-tagged Gce^WT^, Gce^T272Y^, and Gce^V315F^ proteins were generated through conventional *P*-element mediated transformation by injecting embryos of the *w* ^*1118*^ host strain with the *pTFW*-based vectors (Genetic Services, Inc.). In all cases, expression of the transgenic proteins was induced using the Gal4/UAS system [51] with the ubiquitous *armadillo* (*arm-Gal4*) or *α-tubulin* (*tub-Gal4*) drivers (Bloomington *Drosophila* Stock Center, Indiana).

### Genetic rescue experiments

*D*. *melanogaster* with unaffected *Met* ^*+*^ function are sensitive to exogenous JH or its mimics as early prepupae [52], whereas flies deficient for *Met* tolerate exposure to these compounds [20,47]. To test for restoration of methoprene sensitivity to *Met* ^*27*^ mutants, homozygous *Met* ^*27*^; *arm-Gal4* females were mated with *Met* ^*27*^ */Y*; *UAS-gce* or *Met* ^*27*^ */Y*; *UAS-Met* males carrying the wild-type or mutated transgenes, all inserted into the same *attP2* landing site [44]. The uniform *Met* ^*27*^; *arm-Gal4/+*; *UAS-gce (or UAS-Met)/+* F1 progeny was exposed to methoprene, pyriproxyfen or ethanol alone from the outset of larval feeding, and numbers of emerged adults were scored relative to all animals forming pupae.

To test for rescue of viability in the non-conditionally lethal *Met* ^*27*^ *gce* ^*2*.*5k*^ double mutants, balanced *Met* ^*27*^ *gce* ^*2*.*5k*^ */FM7c*; *arm-Gal4* or *Met* ^*27*^ *gce* ^*2*.*5k*^ */FM7c*; *tub-Gal4* females were crossed with *w* ^*1118*^ */Y*; *UAS-gce* or *w* ^*1118*^ */Y*; *UAS-Met* males harboring the wild-type or mutated *gce* or *Met* transgenes in the *attP2* landing site. To detect the transgenic Gce proteins, we used males with *UAS-FLAG-gce* transgenes carried on the *pTFW* vector and inserted into random genomic loci.

### Immunoblotting and antibody tissue staining

Immunoblots were prepared from total *D*. *melanogaster* S2 cell lysates or from entire adult transgenic flies and processed with an anti-FLAG antibody (Sigma-Aldrich; 1:4000) and with anti-Mbf1 or anti-Cheerio antibodies as previously described [53]. Clones overexpressing WT or mutant Gce proteins were induced using the heat-shock-FLPout technique [54], whereby *y w hs-flp*; *act>y+>Gal4*, *UAS-GFP* females were mated to *UAS-FLAG-gce* transgenic males. Fat bodies dissected from larval progeny one day after heat shock (37°C, 30 min) were stained with anti-FLAG (Sigma-Aldrich; 1:1000) and Cy3-conjugated (Cell Signaling) antibodies, and images were captured with the Olympus FV1000 confocal microscope.

## Supporting Information

S1 Fig *Drosophila* S2 cells express endogenous *Met*, *gce*, and *tai*. Reverse transcription of total RNA followed by quantitative PCR revealed expression of endogenous mRNAs encoding Met, Gce and Tai proteins in *D*. *melanogaster* S2 cells. Addition of 1 μM JH III had no appreciable effect on expression of these three genes. The transcript levels were normalized to levels of mRNA for the ribosomal protein 49 (*rp49*). Data are mean ± SD (n = 3).
(TIF)

S2 Fig Transcriptional activation by pyriproxyfen in *Drosophila* S2 cells requires Gce and Tai. The JHRE-luc reporter was induced by 1 μM pyriproxyfen relative to basal activity (ethanol, values arbitrarily set to 1). RNAi-mediated depletion of the endogenous Gce and Tai proteins prevented the induction, whereas *Met* RNAi did not. *egfp* dsRNA served for control. Overexpression of Tai enhanced the pyriproxyfen- and Gce-dependent activation (bottom graph). Data were normalized to *Renilla* luciferase activity and plotted as mean ± SD, representing three independent replicates.
(TIF)

S3 Fig The capacity of Gce to bind JH III is lost upon mutation of specific amino acid residues in its ligand-binding pocket within the PAS-B domain. Gce protein variants were transcribed/translated *in vitro* (reticulocyte lysate) and subjected to the dextran-coated charcoal assay with [^3^H]JH III. Mock, reticulocyte lysate without Gce. Data are mean ± SD (n = 5).
(TIF)

S4 Fig The JH-binding capacity of Gce is required to restore sensitivity to pyriproxyfen in *Drosophila Met* ^*27*^ mutants. *Met* ^*27*^ */Y* males with *UAS-gce* constructs integrated into the *attP2* chromosomal landing site or no transgene (first column) were mated to *Met* ^*27*^ *; arm-Gal4* females, and the F1 progeny was fed on a diet supplemented with pyriproxyfen (5 μg per vial) or solvent (ethanol) alone. About one-third of *Met* ^*27*^ flies survived a dose of pyriproxyfen that was lethal for the same *Met* ^*27*^ strain expressing Gce^WT^ but none of its mutated variants incapable of binding JH. Values are per cent average numbers of emerged adults relative to total number of pupated animals. The total numbers of animals counted in three independent trials are above columns.
(TIF)

S1 Table Primer sets for cloning of cDNA fragments for dsRNA synthesis. (DOC)

S2 Table Primer sets for quantitative reverse transcription-PCR (qRT-PCR). (DOC)
