# Supplementary figures and images for: Genetic Evidence for Function of the bHLH-PAS Protein Gce/Met As a Juvenile Hormone Receptor
Source: PLoS Genet. 2015 Jul 10;11(7):e1005394. doi: 10.1371/journal.pgen.1005394 (PMC4498814; doi:10.1371/journal.pgen.1005394)

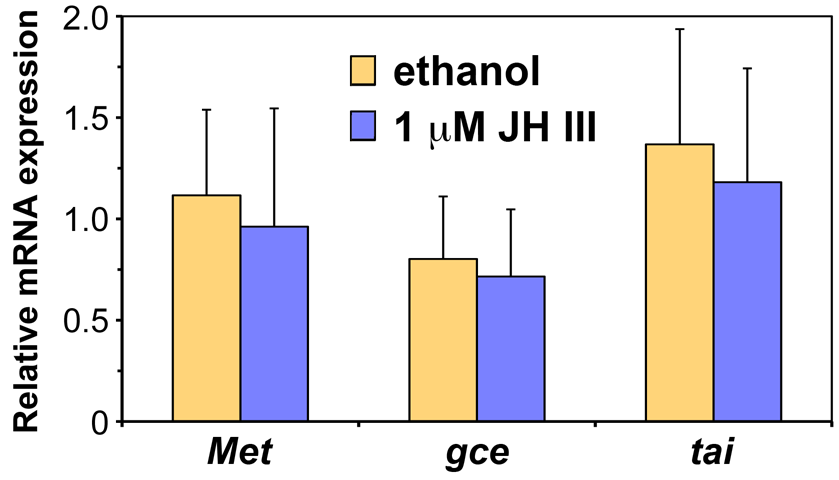

Supplement: S1 Fig — Reverse transcription of total RNA followed by quantitative PCR revealed expression of endogenous mRNAs encoding Met, Gce and Tai proteins in D. melanogaster S2 cells. Addition of 1 μM JH III had no appreciable effect on expression of these three genes. The transcript levels were normalized to levels of mRNA for the ribosomal protein 49 (rp49). Data are mean ± SD (n = 3). (TIF) [file pgen.1005394.s001.tif]

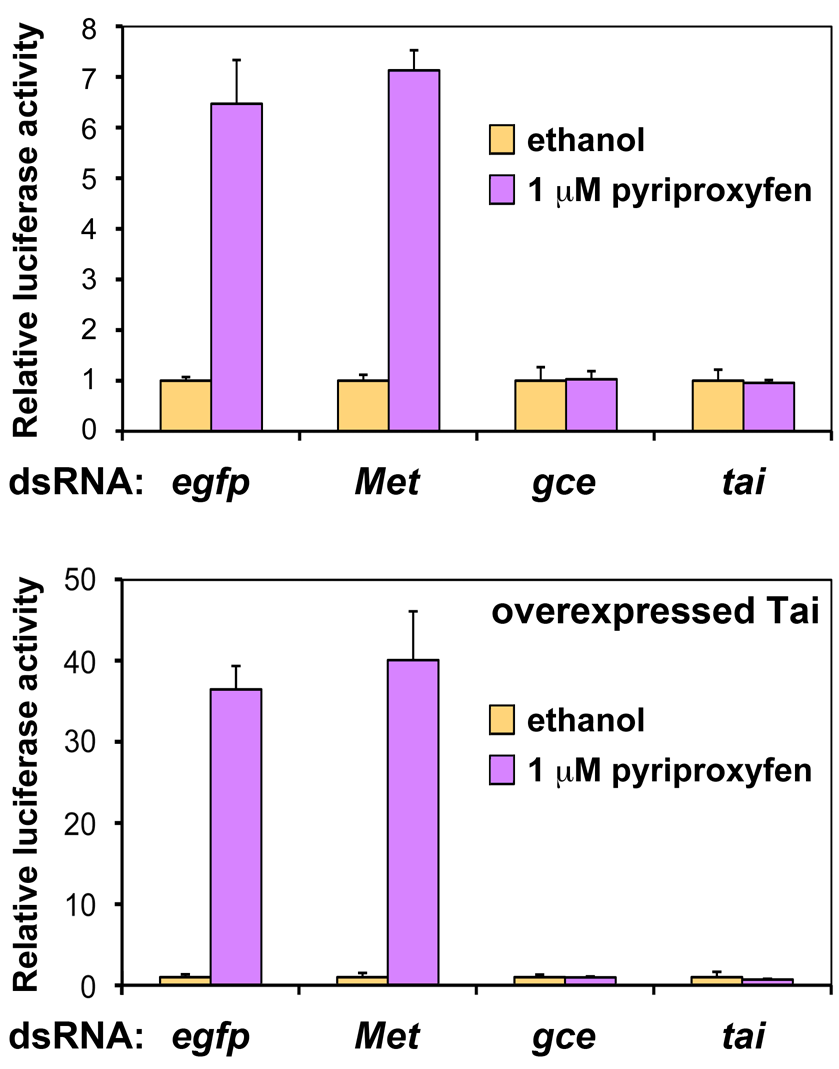

Supplement: S2 Fig — The JHRE-luc reporter was induced by 1 μM pyriproxyfen relative to basal activity (ethanol, values arbitrarily set to 1). RNAi-mediated depletion of the endogenous Gce and Tai proteins prevented the induction, whereas Met RNAi did not. egfp dsRNA served for control. Overexpression of Tai enhanced the pyriproxyfen- and Gce-dependent activation (bottom graph). Data were normalized to Renilla luciferase activity and plotted as mean ± SD, representing three independent replicates. (TIF) [file pgen.1005394.s002.tif]

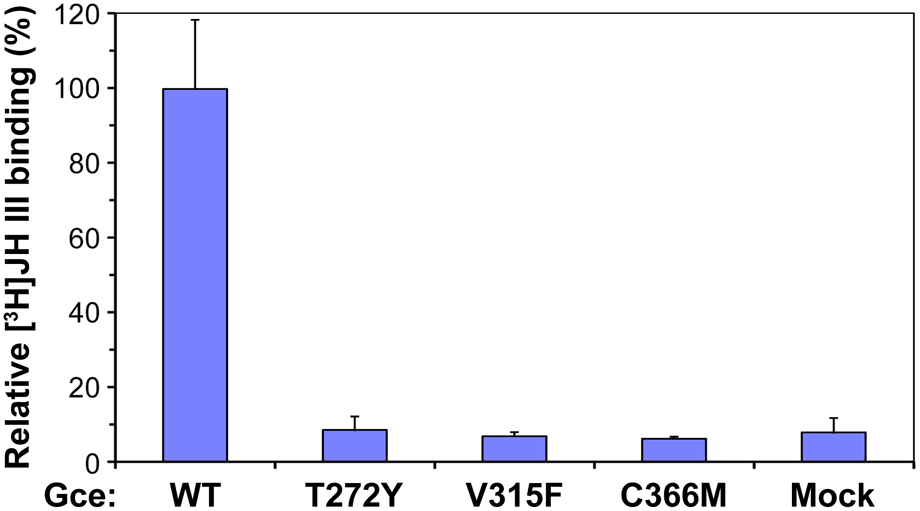

Supplement: S3 Fig — Gce protein variants were transcribed/translated in vitro (reticulocyte lysate) and subjected to the dextran-coated charcoal assay with [3H]JH III. Mock, reticulocyte lysate without Gce. Data are mean ± SD (n = 5). (TIF) [file pgen.1005394.s003.tif]

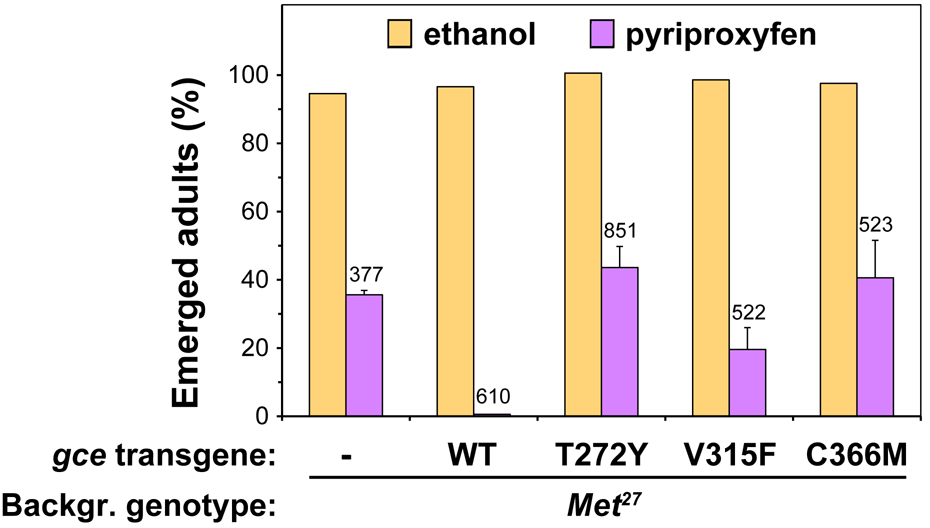

Supplement: S4 Fig — Met 27 /Y males with UAS-gce constructs integrated into the attP2 chromosomal landing site or no transgene (first column) were mated to Met 27 ; arm-Gal4 females, and the F1 progeny was fed on a diet supplemented with pyriproxyfen (5 μg per vial) or solvent (ethanol) alone. About one-third of Met 27 flies survived a dose of pyriproxyfen that was lethal for the same Met 27 strain expressing GceWT but none of its mutated variants incapable of binding JH. Values are per cent average numbers of emerged adults relative to total number of pupated animals. The total numbers of animals counted in three independent trials are above columns. (TIF) [file pgen.1005394.s004.tif]
