# Supplementary material for: Genetic Evidence for Function of the bHLH-PAS Protein Gce/Met As a Juvenile Hormone Receptor
Source: PLoS Genet. 2015 Jul 10;11(7):e1005394. doi: 10.1371/journal.pgen.1005394 (PMC4498814; doi:10.1371/journal.pgen.1005394)
Supplement: S1 Table — (DOC) [file pgen.1005394.s005.doc]

**Table S1. Primer sets for cloning of cDNA fragments for dsRNA synthesis**.

|  | Forward primer (5'-3') | Reverse primer (5'-3') | dsRNA length |
| --- | --- | --- | --- |
| *Met* | CCACGCAGAGCAGCAGTCC | GGTGGCGGTGATGTGTTG | 505 bp |
| *gce* | TCTCAAGCAAGACATTCCCTAC | TGGTTCCTCCTGGCACTC | 563 bp |
| *tai* | GCTGCCGCACAAACGGCAAA | CTCGAGATCGCCCCACACGC | 879 bp |
