# Supplementary material for: Genetic Evidence for Function of the bHLH-PAS Protein Gce/Met As a Juvenile Hormone Receptor
Source: PLoS Genet. 2015 Jul 10;11(7):e1005394. doi: 10.1371/journal.pgen.1005394 (PMC4498814; doi:10.1371/journal.pgen.1005394)
Supplement: S2 Table — (DOC) [file pgen.1005394.s006.doc]

**Table S2. Primer sets for quantitative reverse transcription-PCR (qRT-PCR)**.

|  | Forward primer (5'-3') | Reverse primer (5'-3') |
| --- | --- | --- |
| *Met* | CTGCTTCCTCACCCTAACCT | CGTCTCCATTTCCTCCTCCT |
| *gce* | CGACAAGGAGACGAACAAGG | GTGAGGATAGAGCGGAGGAA |
| *tai* | CGCAAGACGGATTCAAAGGT | GACGCCGCTGATGTAATG |
| *Kr-h1α* | CCGAATACGACATAACAGCC | CGATTTCCGTGAATATGTTCT |
| *rp49* | GAAGAAGCGCACCAAGCACT | CACGTTGTGCACCAGGAACT |
